# Exploring patient acceptability of a short‐stay care pathway in hospital post arthroplasty: A theory‐informed qualitative study

**DOI:** 10.1111/hex.13561

**Published:** 2022-06-30

**Authors:** Cassie E. McDonald, Camille Paynter, Jill J. Francis, Daevyd Rodda, Supreet Bajwa, Dwane Jackson, David Story

**Affiliations:** ^1^ Melbourne School of Health Sciences The University of Melbourne Parkville Victoria Australia; ^2^ Department of Critical Care The University of Melbourne Parkville Victoria Australia; ^3^ Allied Health, Alfred Health Melbourne Victoria Australia; ^4^ Centre for Implementation Research Ottawa Hospital Research Institute Ottawa Ontario Canada; ^5^ Vermont Private Hospital Vermont South Victoria Australia; ^6^ Cabrini Private Hospital Malvern Victoria Australia; ^7^ Sunshine Coast University Private Hospital Birtinya Queensland Australia; ^8^ Buderim Private Hospital Buderim Queensland Australia; ^9^ University of the Sunshine Coast Queensland Australia; ^10^ Sunshine Coast Orthopaedic Group Birtinya Queensland Australia; ^11^ Department of Anaesthesia Royal Brisbane and Women's Hospital Herston Queensland Australia

**Keywords:** acceptability, arthroplasty, hospital, joint replacement, osteoarthritis, short‐stay, theory‐informed

## Abstract

**Introduction:**

Arthroplasty is an effective, yet costly, surgical procedure for end‐stage osteoarthritis. Shorter stays in hospital are being piloted in Australia. In some countries, short stay is established practice, associated with improving perioperative care and enhanced recovery after surgery practices. Exploring the acceptability to patients of a short stay care pathway in hospital postarthroplasty is important for informing health policy, adoption and potential scalability of this model of care.

**Methods:**

Consecutive patients at one site, at least 3 months post total joint arthroplasty, were invited to participate in theory‐informed semi‐structured qualitative interviews. The Theoretical Framework of Acceptability (TFA) informed development of the interview guide. Interview data were analysed using the Framework Method.

**Results:**

Eighteen patients were invited. Fifteen consented to be contacted and were interviewed. Short‐stay post arthroplasty was highly acceptable to patients who had the supports necessary to recover safely at home. Key findings were as follows: flexibility of short‐stay care pathway was essential and valued; prior beliefs and expectations informed acceptability; and the absence of out‐of‐pocket expenses had an incentivizing effect, but was not the primary reason for patients choosing this care pathway. Further themes analysed within the TFA constructs highlighted nuances of acceptability relating to this model of care.

**Conclusions:**

A short stay in hospital post arthroplasty appeared to be acceptable to patients who had experienced this care pathway. Our thematic findings identified aspects of the short‐stay care pathway that enhanced acceptability and some aspects that limited acceptability. These findings can inform refinement of the short‐stay care pathway.

**Patient or Public Contribution:**

Patients/people with lived experience were not involved in the study design or conduct of this preliminary work; as this short‐stay model of care was recently introduced, only a small group of patients was eligible to participate in this study. This study is the first step towards understanding the experiences of patients about a short‐stay model of care post arthroplasty. The findings will help inform future patient and public involvement in expanding the programme.

## 1 INTRODUCTION

Osteoarthritis is a highly prevalent and costly condition affecting more than 500 million people worldwide and is a leading cause of pain and disability in older adults.^1^ Total joint arthroplasty (referred to from herein as arthroplasty) is a common and effective treatment for end‐stage osteoarthritis,^2^ improving both physical function and quality of life.^3^ However, costs of arthroplasty are progressively increasing.^4^ At present, osteoarthritis‐related hospital admissions in Australia cost AUD$1.2billion annually.^3^ Contributing to these costs are initial inpatient admissions as well as readmissions due to postoperative complications.^5^ To address these high costs, safe and effective methods of reducing inpatient length of stay are being investigated.

Advances in perioperative medicine and improvements in patient outcomes after arthroplasty are facilitating gradual reductions in hospital length of stay.^6^ Internationally, length of stay post arthroplasty varies^7^ depending on the preferred model of post operative care. In the United States (US), a short stay in hospital or even same‐day discharge post arthroplasty is established practice.^8^ In contrast, the average length of stay in Australian private hospitals is 4.8 days after total knee replacement (TKR) and 4.9 days after total hip replacement (THR).^9^ Standard care after arthroplasty in Australia typically involves acute hospital care, followed by supervized outpatient or inpatient rehabilitation.^10^ These traditional models of care have been challenged by a growing evidence base demonstrating noninferiority of home‐based programmes for lower‐risk patients after arthroplasty.^11^ While one US study found that post operative length of hospital stay was the least important consideration for patients contemplating arthroplasty,^12^ the preferences and perceptions of short‐stay after arthroplasty amongst Australian patients are unknown.

Adoption of healthcare interventions depends in part on their ‘acceptability’ to intervention recipients.^13^ Patients are more likely to engage with an intervention if they consider it to be acceptable.^13^ According to the Theoretical Framework of Acceptability (TFA), ‘acceptability’ is a multifaceted construct defined as ‘the extent to which people delivering or receiving a healthcare intervention consider it to be appropriate’ (p. 4).^13^ The TFA comprises seven constructs^13^ (Table 1 presents TFA constructs and definitions). The TFA has been used successfully in other (nonsurgical) contexts to identify components that could be addressed to enhance acceptability, such as nurse‐led reviews of inflammatory rheumatological conditions, postnatal exercise and infant feeding practices.^14^, ^15^, ^16^ It has also been applied in a small number of surgical contexts such as maternal–foetal surgery and intravitreal injections for macular degeneration.^17^, ^18^ The TFA has not yet been used in acceptability studies relating to arthroplasty or short‐stay models of care. Given that a short stay in hospital after arthroplasty was a new model of care in Australia, exploring acceptability to patients was important for informing the potential scalability of this care pathway.

**Table 1:** The Theoretical Framework of Acceptability^13^ with definitions adapted for the current study and an additional construct proposed by authors

	Definition of construct
Construct of acceptability	
Affective attitude	How an individual feels about a short stay in hospital after arthroplasty.
Burden	The perceived amount of effort that is required to participate in the short‐stay care pathway.
Ethicality	The extent to which the short‐stay care pathway has good fit with an individual's value system.
Intervention coherence	The extent to which the participant understands the short‐stay care pathway and how it works.
Opportunity costs	The extent to which benefits, profits or values must be given to engage in the short‐stay care pathway.
Perceived effectiveness	The extent to which the short‐stay care pathway is perceived as likely to achieve its purpose.
Self‐efficacy	The participant's confidence that they can perform the behaviour(s) required to participate in the short‐stay care pathway.
Additional construct	
Perceived safety and risk	Any factors perceived to affect safety and risk during the short‐stay care pathway.

## 2 MATERIALS AND METHODS

### 2.1 Study aim

The aim of this study was to explore the acceptability of a short stay in hospital post arthroplasty from the perspective of patients.

### 2.2 Study setting and design

This cross‐sectional, theory‐informed qualitative study was conducted between July and October 2021. The study setting was a single site in Melbourne, Australia, which was the first site where this short‐stay care pathway was routinely offered in the state of Victoria. The researchers approached this study through an interpretivist paradigm, paying attention to people's perspectives in context.^19^ Ethical approval was obtained on 29 July 2021 from the University of Melbourne Office of Research Ethics and Integrity (Ethics Approval ID: 2021‐22186‐20081‐4).

### 2.3 Intervention description: ‘Short‐stay care pathway’ after arthroplasty

**Figure 1:**
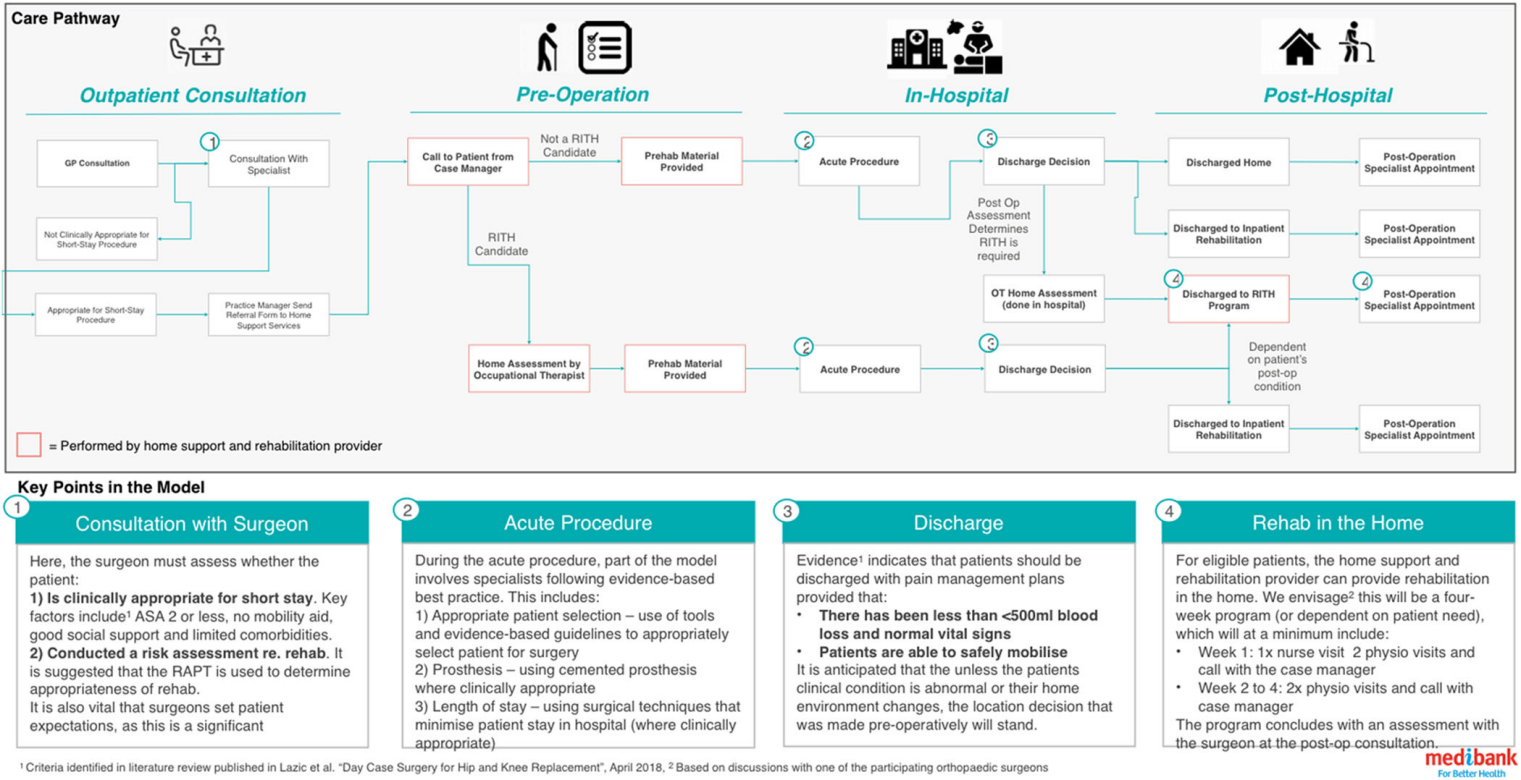
Key stages and processes within the short‐stay care pathway postarthroplasty.

The ‘short‐stay care pathway’ is a complex intervention. A detailed description of the short‐stay care pathway was mapped to the Template for Intervention Description and Replication (TIDieR) checklist^20^ (Supporting Information: File S1). This short‐stay care pathway after arthroplasty was introduced as an initiative of Australian private health insurance companies in partnership with private hospitals and health professionals. The short‐stay care pathway involves four stages: preadmission preparation and information provision; total joint arthroplasty surgery; short stay in hospital; and rehabilitation and recovery at home (Figure 1). The pathway aimed to incentivize appropriate short length of stay for suitable patients and included other measures such as individualized prosthesis selection.

Short stay in hospital typically involves a planned hospital admission of one night or, in some cases, same‐day discharge. The short‐stay care pathway is offered only to eligible patients based on thorough clinical assessment by health professionals. In Australia, another component of the pathway is a ‘no gap’ financial arrangement, whereby the cost of surgery and associated fees (i.e., costs of hospital stay) are set and covered in full by the health insurer.^21^ This means that patients have no ‘out‐of‐pocket’ expenses for their surgery or hospital stay, contrasting with typical out‐of‐pocket doctors’ fees for arthroplasty of AUD$600^22^ (although out‐of‐pocket fees are known to vary and can be up to AUD$10 000). We refer to this arrangement of no added out‐of‐pocket costs as ‘no gap’.

### 2.4 Participant recruitment

Patients who experienced a short stay in hospital after arthroplasty before May 2021 were consecutively identified. At the time of data collection, selecting this date (May 2021) allowed for at least 3 months postsurgical recovery. Patients who were unable to converse in English, or employees of the health insurer (Medibank) or private hospital (Vermont Private Hospital) were not eligible to participate. Eligible patients were contacted by an administrative staff member of the surgical consulting rooms who briefly outlined the aim of the study using a script provided by the research team and who obtained agreement to be contacted by the research team. A member of the research team (C.M. or C.P.) then discussed the study with the patient, provided the patient information and consent forms, explained the reasons for conducting the study, described the interviewer's experience and clinical background and answered any questions. Written or audio‐recorded verbal informed consent was obtained before commencing the interview. Recruitment ceased when the target sample of 15 participants was reached, which was determined by published guidance on sample sizes for theory‐based interview studies^23^ and pragmatic considerations (time and funding).

### 2.5 Data collection

Semi‐structured interviews were conducted using distance modes (seven by telephone, eight by videoconference) to allow for COVID‐safe practice, minimize participant burden and allow recruitment of patients who lived in regional areas. All interviews were conducted by authors C.M. and C.P. (eight and seven interviews, respectively). The interview guide (Supporting Information: Appendix S1) was developed using a systematic process recommended in theory‐informed qualitative research^14^ and informed by the TFA (explained further in the section on ‘Rigor’ below). The interview topic guide was designed to explore the seven constructs of the TFA. The research team proposed an additional construct: ‘perceived safety and risk’ (Table 1). This additional construct was added because the research team (including orthopaedic surgeons, anaesthetists, allied health clinicians and an implementation scientist) posited that ‘perceived safety and risk’ may impact on acceptability in a surgical context and may not be sufficiently covered by the existing TFA constructs.

At the start of each interview, the four stages of the short‐stay care pathway were described, and a visual prompt was given to participants (Supporting Information: Appendix S1). This process ensured a shared understanding between the interviewer and the interviewee of the complex intervention that was the focus of the interview. This process is recommended in theory‐informed qualitative research.^24^ On average, interviews lasted 45 min (range: 35–65 min). Interviews were audio‐recorded and transcribed verbatim. An electronic sociodemographic questionnaire was used to collate participant information at the end of the interviews.

### 2.6 Data analysis

Participants' demographic data were summarized using descriptive statistics. Interview data (audio recordings and transcripts) were analyzed using an inductive, and then a deductive, approach within the Framework Method.^25^ This method was chosen due to its flexibility and because the clear, structured steps can support collaboration between multiple researchers throughout analysis.^25^ The Framework Method as described by Gale et al.^25^ has seven stages: (1) transcription; (2) data familiarization; (3) coding; (4) development of a working analytical framework; (5) application of the analytical framework; (6) charting of data into the framework matrix; and (7) data interpretation. Stages 1–7 were conducted by authors C.M. and C.P. with input from D.S. and J.F. at Stages 4 and 7. A detailed description of the analytical process at each stage is provided in Supporting Information: Appendix S2. NVivo 12 software (QSR International) was used to enhance the organization, management, visualization and reporting of data.

### 2.7 Rigor

The following steps contributed to rigor in the design and conduct of this study. First, the interview questions were ‘back coded’^26^ by experts in implementation science external to the research team. This step checked whether each question aligned with a relevant construct in the TFA. Second, the interview guide was piloted twice to check the coherence, flow, duration and whether the questions elicited appropriate responses. After piloting, the interview guide was condensed, as a few questions were thought to be repetitive and therefore redundant. The interviews were conducted by authors C.M. and C.P., who are clinician researchers (both final‐year PhD candidates at the time) with experience and training in qualitative interviewing techniques. Third, authors maintained an audit trail, during data collection and analysis, of key methodological decisions and study processes. Critical and reflective discussions were held regularly by members of the research team (C.M., C.P., J.F. and D.S.) to debate, review and revise developing categories and themes during analysis, and ensure that thematic interpretations were defensible and strongly linked to the data source. Finally, use of thick description, illustrative quotations and a comparison to existing literature during report writing all supported rigor.^27^ The COnsolidated Criteria for REporting Qualitative research guidelines were considered and, where relevant, addressed during study design, conduct and reporting^28^ (Supporting Information: File S2). Formal member checking was not undertaken due to pragmatics of the study timeframe. Participants were offered a copy of the study results after study closure to communicate key findings.

## 3 RESULTS

Of the 18 patients (6 THR and 12 TKR) invited to participate, 15 consented and were interviewed (eight females, seven males; mean age of 69 years; range: 55–84 years) (Table 2). Three people declined to participate, citing being too busy or declined without reason. Eleven participants underwent TKR, three participants underwent THR and one participant underwent both TKR and THR under the short‐stay care pathway. At the time of the interview, the mean time post surgery was 9 months (range: 3–20 months). Twelve participants were discharged directly home after spending one or two nights in hospital post surgery, and three participants had unplanned discharges to inpatient rehabilitation.

**Table 2:** Participant sociodemographic characteristics

Sociodemographic characteristics	Participants (*n* = 15)
Gender (*n*=)	
Female	8
Male	7
Age, mean (range)	69 years (55–84)
Type of arthroplasty (*n*=)^a^	
THR	4
TKR	12
Time postsurgery mean (range)	9 months (3–20)
Length of stay (*n*=)^b^	
1 Night	6
2 Nights	6
Discharge destination (*n*=)	
Own home	11
Family member's home	1
Inpatient rehabilitation	3
Employment status	
Retired	7
Working full time	6
Working part‐time or casual	2
Health insurance membership (*n*=)	
>50 years	2
25–49 years	5
5–24 years	2
<5 years	6
Living arrangements (*n*=)	
Alone	2
With spouse/family	13

Abbreviations: THR, total hip replacement; TKR, total knee replacement.

^a^ One participant had undergone both THR and TKR.

^b^ Participants admitted to inpatient rehabilitation not included.

Interview results are reported in two sections. Section 1 reports the overarching themes that reflect perceived acceptability of the short‐stay care pathway. Section 2 reports themes that sit within each of the TFA constructs. Participant names accompanying quotes are pseudonyms.

### 3.1 Section 1: Overarching themes of acceptability

The following themes represent findings across the interview data set and reflect perceived acceptability of the short‐stay care pathway.

#### 3.1.1 Flexibility of the short‐stay pathway essential and valued

The flexibility of the short‐stay care pathway was noted by participants as being essential and valued. Many examples where the care plan was modified slightly to suit or respond to their individual needs were shared. Some of the examples indicate small changes to the planned care pathway such as offering additional home cleaning services or relocating physiotherapy appointments to the workplace instead of at home. Other reported examples of changes to the care pathway were substantial. For example, some participants were not able to be discharged home as planned for different reasons.

Adam (TKR): *Because they want me to get out the first night. And I told them, I said, ‘I'm not comfortable enough yet to go home’. And they said, ‘All right. You can stay another night’. …Yeah. The second night, I was comfortable enough [to go home]*.

In these cases, depending on their circumstances, participants either remained in hospital for an additional night and then were discharged home, or were discharged to inpatient rehabilitation.

Marie (TKR): *We're all different and in my case, it probably wouldn't have been an idea to go straight home because of the blanking business [referring to postural hypotensive episodes post‐surgery] that I was having*.

The short‐stay care pathway was occasionally modified according to the needs of the individual following discussions with staff. This flexibility was perceived to influence multiple constructs of acceptability, including how effective they thought it was (perceived effectiveness), how they felt about it (affective attitude) and how much effort was required to participate (burden).

#### 3.1.2 Prior beliefs and expectations influence acceptability

Participants appeared to have strong opinions and prior beliefs about the consequences of home‐based recovery versus inpatient rehabilitation. These opinions and prior beliefs appear to have influenced perceived acceptability of the short‐stay care pathway.

Ryan (TKR): *When you're at home, you can just be fully relaxed…And yeah, I just think that also makes the recovery of the patient a lot, lot quicker and a lot better*.

Adding to these prior beliefs about a short stay in hospital after arthroplasty was how informed a participant was in terms of the potential risks of being admitted to hospital and/or the potential benefits of recovering at home.

Richard (TKR & THR): *So the less time in the hospital probably the better. I understood the process of, the quicker you get back on your feet and get moving, the quicker the recovery*.

Participants' expectations of their recovery from arthroplasty also influenced whether they thought it was acceptable to have a short stay in hospital. An example of this association was where participants described arthroplasty as ‘major surgery’. Participants who perceived arthroplasty as major surgery, but expected that they could recover well at home, thought that a short stay in hospital was acceptable. By contrast, participants who expected that major surgery would require inpatient care to effectively recover did not perceive a short stay in hospital as acceptable.

Rose (TKR): *But also apart from everything else, you're just not well enough to come home … you're just not well enough. Crazy idea*.

Expectations of the short‐stay care pathway were positively influenced by the ‘joint school’ (preoperative education sessions) and preadmission appointments. These information sessions influenced the acceptability of a short stay for participants who were predisposed to the short‐stay pathway. Participants' prior beliefs and expectations of a short stay and recovering at home impacted on many constructs of acceptability, such as perceived effectiveness, safety, affective attitude, ethicality and self‐efficacy.

#### 3.1.3 Incentivizing effect of the ‘no gap’ arrangement

The ‘no gap’ component of the short‐stay care pathway ‘persuades’ people to undertake a short stay in hospital. For some people, the financial arrangement was fundamental to accessing the programme and was therefore highly persuasive.

Rose (TKR): *I was very pleased to be offered this. I thought it was a very good plan… But I was very grateful they did [offer the ‘no gap’] because it meant that I could have my surgery*.

A subgroup of participants changed their health insurance provider to access this programme, which is also indicative of the incentivizing effect of the ‘no gap’ arrangement. Other participants wanted to have a particular surgeon, a particular surgical procedure (i.e., anterior approach) or access a particular hospital (i.e., due to location). (These choices are not available in Australia's publicly‐funded system.) For these participants, the ‘no gap’ arrangement was an important additional incentive.

Thomas (TKR): *But I guess, it's sort of the icing on the cake to find that you don't have to pay extra for sure. No doubt about that…So, I wouldn't say that was the clincher, but it certainly didn't hurt*.

In one negative case, the ‘no gap’ arrangement was not perceived as influencing their decision to proceed with the short‐stay care pathway. This theme illustrates that people engaged with the short‐stay programme for different reasons; however, it reinforces that the ‘no gap’ arrangement was an appreciated and valued incentive. The ‘no gap’ arrangement influenced multiple constructs of acceptability including burden, affective attitude, ethicality and perceived effectiveness.

### 3.2 Section 2: Construct themes

Section 2 reports the themes developed during analysis related to each of the TFA constructs. Brief descriptions are reported below with exemplar quotes for each theme (Table 3). Detailed theme descriptions are provided in Supporting Information: Appendix S3.

**Table 3:** Themes within TFA constructs with exemplar quotes

TFA construct	Construct theme	Exemplar quotes
Perceived effectiveness	Comprehensive ‘package’ with skilled staff at the core	Grace (THR): *So, the whole package: excellent. Really excellent. And if I had a knee done, if I have to have a knee done, I would certainly go back to [this hospital] and go through that process with them again*. Janet (TKR): *All the nursing staff, so the theatre staff, they're so caring and say, ‘It's okay. You're going to be all right’. The anaesthetist just put you at ease. And [the surgeon] has got a beautiful bedside manner*.
	‘Before and after’—outcomes of surgery make it all worthwhile	Thomas (TKR): Yes it certainly has. Very, very effective. So it's helped me a lot…Oh, it was sometimes really simple things like walking up to the local cafe, which I couldn't even walk to the cafe at the end (*referring to pre‐surgery pain). It was so bad that I had to be driven there. Ryan (TKR): *I would want to say, one of the best things I've ever done was having my knee replaced. I've got virtually no pain now, whereas I had a lot constantly all the time*.
	Home‐based care can accelerate recovery	Janet (TKR): *But you just heal better in your own bed and sleep in your own bed and be in your own environment*. Thomas (TKR): *I personally think your state of mind, mental stability, so the mental side of things is much better if you're at home than in hospital. I think if you're mentally better, you're going to heal better quicker anyway*.
Affective attitude	Anxious and trepidatious about unknowns	Paula (TKR): *I was very anxious about the operation and I guess, because of recent events, I was more anxious than I usually am and after I had the operation, it was just a huge relief*. Barney (TKR): *I was a bit worried that I wasn't going to be independent quick enough*.
	Positive feelings during recovery and individualized care	Helen (TKR): *I was happy with the surgeon, happy with the big hospital. … But as far as the nurses go, they were all lovely. And the physio that came home to the house was very nice. And it all went very smoothly*. Ben (TKR): *I couldn't be happier with the whole experience of it. First and second time. The first time I was surprised that it was such a brilliant experience, which I wasn't expecting. And the recovery was quick, which I wasn't expecting*.
Burden	‘No gap’ alleviates the financial burden of private surgery	Rose (TKR): *And she said that the [health insurer] could offer this program. And I said, ‘That sounds great’. Because yes, it really made a difference financially to me*. Sarah (TKR): *I had no concerns for anything financial. And that is a big worry that you don't have when you're dealing with surgery…Yes. Well, I wouldn't take that lightly, the fact that I was arranging to have surgery. That was enough to deal with, let alone financial worries*.
	Managing recovery and advocating for needs	Sarah (TKR): *Burden? No. Well, I mean, the burden was that you had to get up and do these exercises that the physiotherapist insisted on doing every day…But it was not a burden, it was a necessity of, if you want success, you've got to do these things*. Ben (TKR): *I'd just never heard of it before, that the physio would actually come to your house. Because I was thinking, ‘Oh, great, how am I going to get to the physio if I can't drive for four weeks?’*
	Handing over responsibilities to support people at home	Adam (TKR): *I know she [participant's wife] got tired helping me out, but at least she done excellent job…No, the only burden it was for the wife. She had to do everything. Yeah. That's the only burden*. Noah (THR): *Yeah, I think my wife worked quite hard, just filling up the ice containers was, or just even making enough ice …So we had to make all the ice in the house ourselves, so that was quite an effort*.
Opportunity costs	Short‐term reduction in independence and activities	Paula (TKR): *I couldn't drive, that was the biggest thing. I lost my independence for four weeks, but four weeks in the scheme of things, isn't very long at all*.
	Benefits of home‐based recovery	Paula (TKR): *I think if you're in the hospital for a while, you tend to be a bit of a patient…so at home, I was more active and I think that helps in the recovery. It helps with your mental wellbeing about recovery too, I think you're in a much better head space being at home and hobbling around*. Ryan (TKR): *When you're at home, you can just be fully relaxed. And of course, the nurse comes to you, so you're not hopping into the car and getting bounced around or whatever. And yeah, I just think that also makes the recovery of the patient a lot, lot quicker and a lot better*.
Ethicality	Short stay: Effective use of available resources	Richard (TKR & THR): *[Hospital] beds are pretty important, so to me, if I can do the same thing at home and that bed's free for somebody else, it's probably a better thing. Ethical, for my ethical reasons, I'm thinking, ‘Well, if there's not much wrong with me, why am I sitting here on a bed that someone else could have?’ There's that side of it*. Noah (THR): *Well, it could potentially make this surgery much more available to people, because in the public system it's very heavily rationed… I think anything that makes these procedures more available has got to be good*.
	Financial ‘fairness’ and access	Alan (THR): *It [‘no gap’] was fabulous. I can't find a problem with that. If they can find a way of making it easier for people to get this sort of surgery, why not?* Adam (TKR): *Because it's fair for the patient, and fair for the [health insurer]. Because you're paying all the year for insurance there. And if you have to pay extra on the top, what's the use of having the insurance?*
Self‐efficacy	Adequate support to cope physically and emotionally	Helen (TKR): *I had the support of a husband and daughter, and physio. And they all give you confidence*. Adam (TKR): *And I was confident enough. I got out of it…I had the wife. She was helping me all the time. And I had a support, the wife, telling me, ‘You can do it. You can do it’. So, I had support from the family and all*.
	Feeling informed and making progress	Alan (THR): *There's nothing worse than going into the unknown and not knowing what's going to happen, and I think they did it very well out there…So, it was a full education process so that you were pretty confident about what they were doing*. Thomas (TKR): *Well, I was quietly confident, but after I'd been home for days I was completely confident, so. Because as I say, I thought it all went very well*.
Intervention coherence	Variable understanding of the ‘short stay’ in the pathway	Janet (TKR): *There's nothing confusing. It's all streamlined… We'd basically talk it through and they'd send through information. We got sent home with packs and information, so much information. And I felt like I could ring anyone if I didn't understand*. Barney (TKR): *Well, they wanted to bundle me out after 24 h and I protested, but I stayed in there two nights and then I went to [inpatient] rehab [for] 10 days*.
	Knowing the risks and benefits	Thomas (TKR): *Plus the other sort of things like the longer you're in hospital, the more likely you are to get an infection, because it's the most dangerous place you can be is the hospital*. Adam (TKR): *Because as I said to you, I know about hospitals, and I'm old man, and I just didn't want to stay at the hospital and get an infections or anything else*.
Perceived safety and risk	Critical timepoints for clinical and safety assessments	Sarah (TKR): *I had the GP just across the road from me, because a nurse didn't show up, who was supposed to. So I ended up going to my GP, and they took the dressing off my surgery, and checked it out, and redressed it. And yeah, it was after that, then the blood clot developed*. Paula (TKR): *Well, for me it [going home] was fine, but I think these things or medical procedures or anything medical is very much a one‐to‐one, a doctor and a patient decision…Yeah, well [they] assessed me as able to, then very probable I'd be capable of going home early and I think his assessment was accurate*.
	Support at home enhanced safety	Janet (TKR): *I think it's extremely safe as long as you have… Well, they're not going to discharge you if you're struggling, but if you have someone to help you, yes, definitely*. Paula (TKR): *I think because the physio was coming in twice a week. Any concerns, I could always to talk to him, they're specialists in the field these physios. If there was anything going pear‐shaped, I had confidence in him to be able to recognize it*.

Abbreviations: TFA, Theoretical Framework of Acceptability; THR, total hip replacement; TKR, total knee replacement.

#### 3.2.1 Perceived effectiveness

Three themes demonstrated that perceived effectiveness was a strong driver of acceptability for this intervention: Theme 1: *Comprehensive* ‘package’ *of care with skilled staff at the core* was strongly linked to perceived effectiveness. Theme 2: *Before and after—outcomes of surgery make it all worthwhile* indicated that the positive clinical outcomes after arthroplasty were reported as a primary reason for perceiving the surgery and recovery as worthwhile. Theme 3: *Home‐based care can accelerate recovery* highlighted that recovering in a comfortable and familiar environment contributed to perceptions of the short‐stay model of care as effective.

#### 3.2.2 Affective attitude

Theme 1: *Anxious and trepidatious about unknowns*, participants felt anxious or apprehensive before surgery and discharge home because they wondered if they would recover and manage well. Theme 2: *Positive feelings during recovery and individualized care* were expressed by participants.

#### 3.2.3 Burden

Theme 1: ‘No gap’ *alleviates financial burden*. Participants described ‘burden’ in this context as relating to both financial costs and emotional worry. Theme 2: *Managing recovery and advocating for needs* represents participants' acknowledgement that some effort was required to manage their recovery at home and (on occasions) to advocate for their needs. Theme 3: *Handing over responsibilities to support people at home* represented the burden that fell to carers and support people.

#### 3.2.4 Opportunity costs

Theme 1: *Short‐term reduction in participants' independence and activities*, which was anticipated during recovery from surgery and therefore did not detract from the acceptability of the care pathway overall. Theme 2: *Benefits of home‐based recovery* such as less travel time (e.g., to outpatient appointments) and sleeping better in their own bed were highlighted by participants who recovered at home.

#### 3.2.5 Ethicality

Theme 1: *Effective use of available resources* was a notion identified by participants who perceived that a short stay in hospital aligned well with their values as it would ‘free up’ hospital beds for people who needed them and thus represented an effective use of finite healthcare resources. Theme 2: *Financial* ‘fairness’ *and access* was attributed to the ‘no gap’ arrangement. Participants believed that this ‘fair’ arrangement allowed access to private surgery for some people who otherwise may be unable to afford the out‐of‐pocket fees typically incurred with private surgery.

#### 3.2.6 Self‐efficacy

Theme 1: *Adequate support to cope physically and emotionally* at home influenced participants' confidence and whether they felt able to and/or did cope at home. Theme 2: *Feeling informed and making progress* were described by participants who felt informed following consultations with health professionals, which instilled confidence about their surgery and recovery. When participants perceived that they were making ‘good’ progress after surgery, this further contributed to a sense of self‐confidence to manage at home.

#### 3.2.7 Intervention coherence

Theme 1: *Variable understanding of the* ‘short stay’ *in the pathway* was identified, including differing understanding of the estimated hospital stay duration and what recovery at home would involve. Some participants felt well informed, whereas others felt surprised and unprepared for discharge after one night in hospital. In contrast, participants clearly articulated a strong understanding of the arthroplasty procedure and the ‘no gap’ arrangement. Theme 2: *Knowing the risks and benefits* of inpatient versus home‐based recovery influenced acceptability of the short‐stay care pathway. Participants who recounted knowledge of the potential risks of staying in hospital (i.e., developing an infection) explained that this was an important factor driving their preference to recover at home.

#### 3.2.8 Perceived safety and risk

Theme 1: *Critical timepoints for clinical and safety assessments* were highlighted by participants. Participants perceived that thorough clinical monitoring and assessment was critical to their safety, especially during the early phases of recovery at home. This theme was supported by examples shared by four participants who developed postoperative complications at home (wound infections *n* = 2, lower limb blood clots *n* = 2), who stated that they felt these complications may have been detected earlier with closer monitoring. Theme 2: *Support at home enhanced safety* reflected participants' perceptions that sufficient support at home enhanced their safety after discharge.

## 4 DISCUSSION

This Australian study explored the acceptability of a short‐stay care pathway post arthroplasty from the perspectives of patients. We found that this pathway was highly acceptable for patients who had been carefully assessed and had the supports necessary to recover safely at home. As the more detailed results (Supporting information: Appendix S3) demonstrate, the thematic findings identified aspects of the short‐stay care pathway that enhanced acceptability and some aspects that limited acceptability. These findings can inform refinement of the short‐stay care pathway by clinicians, hospital administrators and health insurers to benefit future patients.

Flexibility within a short‐stay model of care was seen as essential for acceptability from patient perspectives. Patients highlighted examples of flexibility at each stage, for example, changes to discharge plans, tailored home services and individualized home‐based rehabilitation. Ultimately, staff, in consultation with patients, need to have the ability to adapt patient care plans as indicated and this needs to be factored into the design of short‐stay care pathways. Although investigation of operational and budget capacity was beyond the scope of this study, we posit that designing flexible business cases and budgets for short‐stay models of care may be important for facilitating the flexibility described by patients in our study.

The comprehensive ‘package’ of care offered within the short‐stay care pathway was associated with high levels of acceptability. It is not only the ‘short stay’ in hospital that was acceptable but also other factors such as the ‘no gap’ financial arrangement, skilled and caring staff and detailed preoperative information sessions that fed into overall acceptability. Financially, the ‘no gap’ arrangement was perceived as essential by some participants and as a bonus by others, and thus, its influence on acceptability varied. This suggests that the influence of a ‘no gap’ arrangement on acceptability may depend on patients' financial circumstances, which may have implications for the scalability of this intervention. Detailed information sessions before surgery are a hallmark of this model of care. Participants indicated that they valued feeling informed and that preoperative information sessions reduced feelings of anxiety and apprehension. These findings contrast with a Cochrane review from 2014^29^ that found that preoperative education offered only a small beneficial effect on preoperative anxiety for THR patients (no data were available for TKR patients). A recommendation of the review was that the efficacy of preoperative education be improved, for example, by tailoring education to the individual.^29^ Potentially, the findings from our study indicate that preoperative education in the short‐stay care pathway (which involved both individual and group sessions) may have been effective in reducing preoperative anxiety.

Outcomes of the surgery itself (i.e., reduced pain, increased mobility) were integral to perceived effectiveness of the short‐stay care pathway for participants. This is similar to findings of a study with patients after pilonidal sinus surgery, whereby acceptability was associated with recovery outcomes.^30^ In contrast, one TFA‐based study with parent participants who underwent maternal–foetal surgery for spina bifida found that the outcomes were not associated with acceptability.^17^ In this context, parents felt responsible to try ‘anything in their power’ and so the high‐risk intervention was perceived as acceptable even when the post surgical outcomes were disappointing (p. 910).^17^ This suggests that the clinical outcomes of surgery may not always drive acceptability; however, in the context of arthroplasty within a short‐stay model of care, post operative outcomes appear to be important for acceptability.

### 4.1 Implications for practice

Issues related to perceived safety during the early phases of recovery at home were identified and affected acceptability. Some participants identified early signs of postoperative complications soon after discharge (i.e., swelling, wound ooze, increasing pain), which they thought did not receive timely review as per the planned pathway. In most of these cases, participants were resourceful and initiated review with their own general practitioner. These cases indicate that changes in postoperative surveillance and intervention may further increase acceptability.

As these findings also suggest that in surgical contexts perceived safety and risk may impact acceptability, other researchers using the TFA may wish to consider including the construct of ‘perceived safety and risk’. Further investigation as to whether perceived safety and risk impacts on acceptability of interventions in other surgical and nonsurgical contexts is warranted.

Further opportunities for improving acceptability of the short‐stay care pathway were identified in relation to holistic preoperative screening. Adequate support at home to be able to manage was important to participants. Adequate support included a capable caregiver or support person able to provide physical assistance with activities of daily living and, in some cases, emotional support. This indicated that closer, comprehensive preoperative screening of patients' living arrangements and the level of support available at home may benefit acceptability. At least one participant in our sample strongly believed that recovery at home was unsafe and that inpatient rehabilitation would aid their recovery. In an Australian study exploring the acceptability of different modes of rehabilitation, Buhagiar et al.^31^ also found that positive past experiences and beliefs about level of support influenced patients' rehabilitation preferences. Preoperative screening of patients' beliefs and expectations about in‐hospital versus at‐home recovery may identify patients for whom a short‐stay care pathway may be unsuitable.

Participants' understanding of the ‘short stay’ component of the care pathway varied, and uncertainty undermined acceptability. Participants had a strong understanding of the arthroplasty procedure, which suggests that information provided about this stage of the care pathway was comprehensive and generally well understood. In contrast, participants demonstrated variable understanding of the ‘short‐stay’ component. Despite strong evidence that home‐based rehabilitation is safe and effective,^32^ a recent study in the US also found that before surgery, over 70% of patients had safety concerns and therefore did not think that they would be able to undergo arthroplasty as an outpatient.^33^ To address perceived safety concerns, thorough descriptions of ‘short‐stay’ (i.e., expected length of hospital stay, benefits of recovering at home and typical risks associated with longer hospital stays) in preoperative information sessions and checking patients' understanding may enhance acceptability.

As previously described, we used Sekhon et al.'s^13^ definition of acceptability as ‘a multifaceted construct that reflects the extent to which people delivering or receiving a healthcare intervention consider it to be appropriate’ (p. 4). Most of the seven TFA constructs appeared to be associated with acceptability of this short‐stay care pathway, as did our additional newly proposed construct of ‘perceived safety and risk’. Perceived effectiveness of arthroplasty in increasing mobility and decreasing pain may have been a particularly strong driver of retrospective acceptability. However, most of the other constructs (with the possible exception of opportunity costs) also appeared to be associated with acceptability from patient perspectives.

### 4.2 Strengths and limitations

The strengths of this study include use of the TFA to inform the study design. The use of theory‐informed approaches in implementation research is strongly advocated.^34^, ^35^ Acceptability is a complex concept and, until recently, was poorly defined; therefore, use of the TFA is proposed to assist with operationalizing this concept and guiding study methods.^13^ We report several findings on using the TFA in a surgical context and on the adequacy of the TFA in surgical contexts in a separate publication.^36^

Another strength of this study was the initial inductive analysis, which allowed the research team to consider acceptability across the TFA constructs, identify relationships between some constructs and explore factors outside the constructs that might be associated with acceptability.

Although steps were taken to reduce sampling bias (i.e., consecutive sampling), the sample did not include any self‐employed participants and included only two casual or part‐time employed participants. This may limit the range of perceptions obtained particularly related to the opportunity cost construct as participants in our sample did not appear concerned about having to take time off work during their recovery period. Further, only English‐speaking participants were included. Therefore, these findings may not reflect the experiences of patients from culturally and linguistically diverse backgrounds, which warrant further investigation.

Only the perspectives of those who received the intervention were obtained for this study. Implementation success is also proposed to be linked to the perceived acceptability of those *delivering* the intervention.^13^ The perspectives of stakeholders such as orthopaedic surgeons, anaesthetists, hospital managers and rehabilitation‐in‐the‐home professionals warrant consideration in future research. Given that the results have implications for carers, partners and family members of patients postarthroplasty, further research exploring their perspectives may be beneficial.

## 5 CONCLUSION

Patient acceptability of a short stay in hospital postarthroplasty was conditional on a range of factors such as skilled and caring staff, comprehensive care, adequate support to manage at home and perceived positive clinical outcomes post arthroplasty. The addition of a financial arrangement that eliminated out‐of‐pocket expenses incentivized this model of care and contributed to its acceptability for some patients. Potential areas for improving the acceptability of this care pathway were identified. Overall, patients found this short‐stay model of care acceptable.

## CONFLICT OF INTEREST

Research Assistant salaries for Cassie McDonald and Camille Paynter were funded by the research grant originally from the Medibank Better Health Foundation. Daevyd Rodda contributed to the development of the model of care being studied. Daevyd Rodda and Dwane Jackson are financial beneficiaries of surgeries performed under this model of care at Vermont Private Hospital. David Story has received previous research grants from the ANZCA Research Foundation, including some with original funding from the Medibank Better Health Foundation. Jill Francis has received previous research grants from the ANZCA Research Foundation. Other authors have no conflict of interest to declare.

## ETHICS STATEMENT

Ethics approval was granted by the University of Melbourne Office of Research Ethics and Integrity (Approval ID: 2021‐22186‐20081‐4).

## Supporting information

Supporting information.

Supporting information.

Supporting information.

## Data Availability

The data that support the findings of this study are not available on request or publicly due to privacy and ethical restrictions.
