# Supplementary material for: Exploring patient acceptability of a short‐stay care pathway in hospital post arthroplasty: A theory‐informed qualitative study
Source: Health Expect. 2022 Jun 30;25(4):2002–14. doi: 10.1111/hex.13561 (PMC9327831; doi:10.1111/hex.13561)
Supplement: Supplementary file 1 — Supporting information. [file HEX-25--s001.docx]

**Appendix 1**

**Interview Guide**

The interview guide included an introductory script which described the ‘joint replacement experience’ and what is meant by ‘short stay in hospital’.

| ***Warm up*** | Can you talk me through how you were offered the short-stay care pathway for your joint replacement?  How long did you stay in hospital (how many nights)?  How has your recovery been? |
| --- | --- |
| ***Affective Attitude:***  *How an individual feels about the intervention, after taking part* | How did you feel during your joint replacement experience?    ***Prompts:***  *Before your surgery?*  *During your stay in hospital?*  *During your recovery at home?*    *[listen for words about discomfort, pain, anxiety etc; may need to probe for more information with appropriate reflective listening]* |
| ***Burden:***  *The amount of effort that was required to participate in the intervention* | We realise that the ‘no gap’ component of your surgery is meant to ensure that you do not incur any ‘out-of-pocket’ expenses. Were there any unexpected financial costs you had to pay before or after your joint replacement?  ***Prompt:****Were these costs unexpected?*    Did you experience any (other) burden(s) because of your joint replacement?    ***Optional (if not yet addressed):*** *What about other members of your household?*  *Clarify details / supports provided as needed* |
| ***Perceived Effectiveness:***  *The extent to which the intervention is perceived to have achieved its intended purpose* | Do you think your joint replacement has been effective for you?    What weren’t you able to do prior to your joint replacement that was important to you? Are you able to do this now? |
| ***Intervention Coherence:***  *The extent to which the participant understands the intervention and how it works* | You told me before that stayed xx nights in hospital…    Some health professionals think that it may be good for patients to have such a short stay in hospital. What do you think about that? |
| ***Opportunity Cost:***  *Experienced opportunity cost: The benefits, profits or values that were given up to engage in the intervention* | Was there anything that you had to give up so that you could have your joint replacement surgery?    ***Prompts:***  *Before your surgery?*  *During your stay in hospital?*  *During your recovery at home?* |
| ***Self-efficacy:***  *The participant’s confidence that they can perform the behaviour(s) required to participate in the intervention* | How confident were you that you could safely recover and complete your rehabilitation program at home? |
| ***Ethicality:***  *The extent to which the intervention has good fit with an individual’s value system* | Do you think there are any ethical issues with offering a short stay in hospital after joint replacement?    And the ‘No gap’ aspect, do you think there are ethical issues with that?    ***Prompt:****In what ways do you think having a short stay in hospital after joint replacement surgery is fair or not fair?* |
| ***Perceived Safety:***  *Any factors perceived to affect safety and risk during the intervention* | Based on your experience, how safe do you think it is to go home after a short stay in hospital following joint replacement surgery?    ***Prompt:****Were there any risks that you were concerned about?* |
| ***Perceived Quality of Care:***  *How an individual perceived the quality of care they received* | Can you comment on the quality of care you received during your joint replacement experience? |
| ***‘No gap’ and ‘short-stay’ comparison*** | Do you have any comments about the ‘no gap’ component of your joint replacement?  ***Prompt:****How important was the promise of ‘no gap’ to you?*  *Compared with the ‘no gap’, how important was the short stay in hospital to you?* |
| ***Optional questions*** | *OPTIONAL: If participants have not explicitly referred to the support services*  *Do you have any comments about the nursing or physiotherapy support services you received at home?* |
| ***Closing*** | Thank you for giving me lots of fantastic information. Can I ask you now to think about overall, when considering all the things you’ve spoken about, what do you think about your joint replacement experience?    Is there anything that you think could be done better?    Is there anything else you’d like to tell us? |
| ***General probes*** | *Can you tell me more about that?  How did you feel about that?*  *That’s interesting*  *Can you say more about that?*  *Can you give an example?* |

**After interview:**

- Stop recording
- Thank participant for their time
- Assist with completing electronic demographic questionnaire if required
- Confirm demographic information obtained
- Offer participant a copy of the results when complete

**Interview Visual Prompt**

The visual prompt was developed to aid participants’ reflections on the whole pathway (not just the surgical procedure) and their experiences at different timepoints (i.e., during their hospital stay, and post discharge during their recovery at home).

**The key stages of your ‘joint replacement experience’ include:**


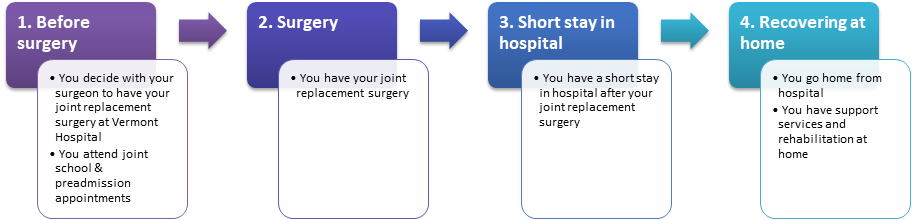


**When we say ‘short stay in hospital’ we are referring to:**


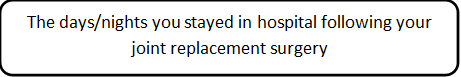


**Appendix 2**

**Details of analytical procedures during stages 1-7 of Framework Method**

*Stage 1 Transcription*

Verbatim transcription of interview recordings were conducted by an external provider. All transcriptions were checked against audio recordings for accuracy and de-identified.

*Stage 2 Familiarisation with data*

Data familiarisation involved reading interview transcripts and listening to audio-recordings multiple times.

*Stage 3 Coding*

An inductive coding approach was used across the dataset during open coding. Line-by-line coding was conducted by CM and CP on 6 interviews (3 interviews each) which generated hundreds of codes. Stage 3 onwards were completed in NVivo software to assist with the organisation, management, visualisation, and reporting of data.

*Stage 4 Developing a working analytical framework*

A rigorous review of all codes was undertaken by CM and CP to develop a working analytical framework. Rich discussion between CM and CP achieved consensus regarding codes to include and generated clear definitions for each code. Through this process, overlapping codes were condensed and extraneous codes removed. The TFA constructs were then added to the working analytical framework. Some of the inductive codes developed in stage 3 aligned with the TFA constructs so these were organised accordingly within the working analytical framework. Inductive codes which did not fit within TFA constructs were organised into separate categories. As recommended by Gale et al (2013) codes were assigned numbers (for easy identification during the coding process) and an ‘other’ category was added to the working analytical framework in case new ideas (which did not fit within the working analytical framework) were identified during later stages of analysis. A “good quotes” code was also included in the working analytical framework to facilitate ease of reporting results.

*Stage 5 Applying the analytical framework*

The working analytical framework (i.e., the inductive and deductive coding structure) was applied to the whole dataset. This involved coding all interview transcripts; phrases, sentences, and paragraphs were assigned to codes.

*Stage 6 Charting the data into the framework matrix*

Data were charted into a framework matrix. The matrix organises data (coded transcripts) into columns (codes) and rows (participants). NVivo software generated the framework matrix which was exported into Excel for charting. Charting involves a delicate balance of summarising (or abstracting) the data without removing the original meaning. Links or references to ‘good quotes’ were included in the matrix.

*Stage 7 Interpreting the data*

The final stage of interpreting the data was iterative and recursive. Data were considered within and across columns and rows of the matric to develop themes. Theme descriptions were developed, revised and exemplar participant quotes were selected.

**Appendix 3**

**Perceived effectiveness**

*Comprehensive ‘package’ with skilled staff at the core*

Participant’s perceived effectiveness of the short-stay care pathway was attributed to the thoughtful and comprehensive ‘package’ of care offered throughout the short-stay care pathway from pre-admission through to home-based recovery. At the core of perceived effectiveness was the perception of staff as skilled, caring and attentive. Positive interactions and relationships with staff were strongly linked by participants to the effectiveness of the short-stay care pathway. Participants raised this in response to multiple interview questions. This theme has some overlap with the construct of affective attitude as perceptions of staff care contributed to many positive feelings associated with the short-stay care pathway.

*‘Before and after’ – outcomes of surgery make it all worthwhile*

Positive outcomes of arthroplasty (i.e., less joint pain, better mobility, and returning to favourite leisure activities) were reported by participants as a primary reason for perceiving the surgery and recovery to be effective and worthwhile. Participants shared many examples where they compared their knee ‘before and after’ to illustrate how effective the surgery had been for them. This theme indicates that participants focussed heavily on the outcomes of surgery when reflecting on their perceptions of the effectiveness of the short-stay care pathway.

*Home-based care can accelerate recovery*

Home-based recovery in a comfortable familiar environment can ‘accelerate’ recovery for some participants. Positive aspects of being at home which were highlighted as contributing to faster recovery, included: sleeping in one’s own bed, being active within a familiar environment, and having support people around. Some participants highlighted that the opportunity to recover at home was central to their decision to proceed with their arthroplasty within this model of care. However, a couple of participants doubted the efficacy of recovering at home and therefore the word ‘can’ is used in the theme name to indicate these hesitations.

**Affective attitude**

*Anxious and trepidatious about unknowns*

Participants frequently described feeling anxious or apprehensive prior to their surgery and prior to discharge home. When participants described these feelings, they were associated with the ‘unknowns’ of undergoing surgery via a short-stay care pathway. ‘Unknowns’ included wondering if: they would manage at home after discharge; in what timeframe would they return to work; and when they might return to their usual activities. The level and extent of anxiety felt by participants varied. The few examples where participants described no anxiety at these timepoints was ascribed by them to feeling very well informed about the surgery and recovery process.

*Positive feelings during recovery and individualized care*

Participants expressed many positive feelings when reflecting on: 1) the care they received from staff at each stage of care pathway; 2) the no gap arrangement; and 3) due to the positive outcomes of their arthroplasty. Feeling happy with their experience was strongly linked to perceiving their care as individualised and tailored to their needs. Feeling happy about their care and recovery was not related to having a short stay in hospital and recovering at home for some participants. However, others felt happy because they were able to negotiate inpatient rehabilitation and perceived this as essential to their successful recovery.

**Burden**

*‘No gap’ alleviates financial burden of private surgery*

The financial ‘no gap’ arrangement was indicated as a substantial alleviator of burden for patients. The promise of ‘no gap’ also reduced the emotional burden associated with worrying about high or unexpected out-of-pocket costs after the operation. Participants described how the ‘no gap’ arrangement reduced their perceived financial burden which in turn reduced their feelings of worry or concern. Therefore, this finding has some overlap with affective attitude.

*Managing recovery and advocating for needs*

Participants stated that effort was required to: 1) manage their physical limitations during the early phases of post-operative recovery; and 2) actively participate in rehabilitation at home. Participants shared that effort was required on occasions to advocate to staff for their needs if a component of their care was not satisfactory. Actively participating in recovery at home included completing an exercise program and preparing ice daily for the ice machine. However, this effort was not always perceived as a burden. For example, participants often described the effort of participating in an exercise program at home as an accepted and ‘normal’ part of recovery from arthroplasty.

*Handing over responsibilities to support people at home*

This theme represents the effort required of carers and support people at home. Participants described a process of ‘handing over’ some of their responsibilities during the early phases of their recovery at home because they needed help with activities of daily living. Participants acknowledged that these burdens for their carer or support person were short-term. The concept of ‘handing over’ in this theme also acknowledges that many of responsibilities previously managed by inpatient health professional staff (i.e., wound care, daily rehabilitation sessions, self-care, and domestic activities) are now ‘handed over’ and managed in the home.

**Opportunity costs**

*Short-term reduction in independence and activities*

Participants reflected they had reduced independence for a brief period with activities such as driving, walking outdoors, and leisure activities whilst physically recovering from the operation. These reflections were mostly shared following prompting from the researchers during interviews, that is, they were not spontaneously shared. This suggests that participants may not have perceived the short-term restrictions they experienced as ‘giving things up’ (opportunity costs). Rather they appeared to see these as temporary limitations which were expected during recovery from arthroplasty. Many of these temporary limitations were in relation to their arthroplasty, not specifically due to participating in the short-stay care pathway.

*Benefits of home-based recovery*

Participants who experienced a short stay in hospital and were discharged directly home perceived that there were benefits from recovering at home. This theme overlaps with perceived effectiveness (‘home-based care can accelerate recovery’). Participants hypothesised that they would not have experienced these benefits if they had remained as an inpatient for a longer stay in hospital. Benefits of recovering at home reported by participants were: less travel time to appointments; recovering in home environment was relaxing and healing; and they felt motivated to be as self-sufficient as possible with activities of daily living.

**Ethicality**

*Short stay: effective use of available resources*

Some participants reflected on the broader ethical implications of the short-stay care pathway for the health system. They reflected that if they only stayed in hospital as long as absolutely necessary, then their discharge would ‘free up’ the hospital bed for the next person. This theme suggests that the short-stay care pathway may fit well with patients’ values when considering broader health system bed pressures and effective use of available resources.

*Financial “fairness” and access*

Participants reflected on the “fairness” of the ‘no gap’ arrangement. Specifically, that in paying health insurance fees (often for many years) it was reasonable to expect not to pay out-of-pocket fees for their surgery and hospital stay. The ‘no gap’ arrangement was also perceived as ‘fair’ as it offered access to private surgery for some participants who felt they may not have afforded this if out-of-pocket expenses were incurred. Participants shared that in-principle this ‘no gap’ arrangement, the fact that there were no hidden or unexpected costs after surgery, fit with their values even if the financial costs of surgery were not a concern for them personally. Participants raised a ‘caveat’ to the ethicality of this ‘no gap’ arrangement that the standard of care must be maintained. They alluded to some cynicism as to ‘why’ this option is available and ‘who may be benefiting’ from the arrangement; however, this was not seen as an ethical concern so long as patients continued to receive high quality and effective care.

**Self-efficacy**

*Adequate support to cope physically and emotionally*

Participants described their ability to ‘cope’ at home as being in relation to both physical and emotional coping. Adequate support influenced their confidence and whether participants felt able to and/or did cope at home. This theme holds in both directions: when participants had adequate support they felt able to cope at home and when participants did not have adequate support at home they felt they would be unable to cope with a short stay in hospital.

*Feeling informed and making progress*

Participants described how their confidence was associated with feeling informed and making progress during their recovery. When they felt informed following consultations with health professionals in the lead up to their surgery this instilled confidence about their surgery and recovery. After surgery, when participants perceived that they were making ‘good’ progress this further contributed to a sense of self-confidence regarding early discharge from hospital and capability to manage at home. There were exceptions where some participants did not feel confident about early discharge home despite feeling informed. In these cases, participants required inpatient rehabilitation.

**Intervention Coherence**

*Variable understanding of the ‘short stay’ in the pathway*

Participants indicated varying understanding of the estimated duration of their stay in hospital and what would be involved in recovery at home. This theme was identified through explicit comments from participants expressing uncertainty about aspects of the care pathway and tacitly throughout participants’ responses to interview questions. Some participants discussed how they felt well informed about the short-stay care pathway. Others shared examples of how they did not feel informed about aspects of the short stay in hospital, for example, feeling surprised and unprepared for discharge after one night in hospital. In contrast, participants clearly articulated a strong understanding of the arthroplasty procedure and the ‘no gap’ arrangement.

*Knowing the risks and benefits*

Knowledge of the risks and benefits of recovering as an inpatient in hospital versus recovering at home influenced acceptability of the short-stay care pathway. Knowing the risks and benefits of each option was informed by participants’ past health experiences and their health knowledge. Participants’ who recounted their knowledge of the potential risks of staying in hospital (i.e., developing an infection) explained that this was an important factor driving their preference to recover at home.

**Perceived safety and risk**

*Critical timepoints for clinical and safety assessments*

Participants perceived that thorough clinical assessment was critical to their safety at two key timepoints. The first timepoint was prior to discharge home from hospital and the second timepoint was during the early stages of recovery at home. Participants felt that it was safe to continue their recovery at home if staff: conducted thorough assessments, used these assessments to inform their recommendations, and discussed their assessments with them (the patient). The critical importance of clinical assessment in the early stages of recovery was emphasised by examples shared by four participants who developed post-operative complications at home (wound infections n =2, lower limb blood clots n =2). Participants stated that they felt these complications may have been detected earlier with more frequent assessment and this was an area where safety could be improved.

*Support at home enhanced safety*

Participants described how having sufficient support at home enhanced their safety once discharged home. Examples of supports shared by participants included both informal family or carer support and formal support from staff or care services. Participants illustrated that when support at home was not available (i.e., they lived alone or their family members were not able to provide the necessary support) this affected their perceived safety to recover at home.
