# Supplementary material for: Exploring patient acceptability of a short‐stay care pathway in hospital post arthroplasty: A theory‐informed qualitative study
Source: Health Expect. 2022 Jun 30;25(4):2002–14. doi: 10.1111/hex.13561 (PMC9327831; doi:10.1111/hex.13561)
Supplement: Supplementary file 2 — Supporting information. [file HEX-25--s003.docx]

**Supporting Information File 1**

**ASAP Intervention Description**

Below the short-stay care pathway is described in detail and mapped to TIDieR Checklist (Hoffman et al., 2014)

| **Item** | **Description** |  |
| --- | --- | --- |
| 1. **BRIEF NAME** | ***Provide the name or a phrase that describes the intervention*** |  |
|  | - The official name for this program as per the health insurer is the ‘No Gap Joint Replacement Pilot Program’. - In this study we refer to the program as a the ‘short-stay care pathway post arthroplasty’. |  |
| **WHY** | ***Describe any rationale, theory, or goal of the elements essential to the intervention*** |  |
|  | - This complex intervention is a short-stay post arthroplasty model of care. - The aim of this model is to optimise patient functional outcomes and reduce length of stay following total hip arthroplasty (THA) or total knee arthroplasty (TKA) and to discharge patients directly home < 3 days post-surgery. - Early discharge home is possible by enhancing recovery after surgery and transferring part of the in-hospital care and rehabilitation to patients’ home. - This particular short-stay model of care offers a financial benefit to eligible patients; the cost of surgery and associated fees (i.e., costs of hospital stay) is set and covered in full by Medibank. Therefore, patients have no ‘out of pocket’ expenses for their surgery or hospital stay referred to by the health insurer as a ‘no gap’ service. |  |
| **WHAT materials** | ***Materials: Describe any physical or informational materials used in the intervention.***  ***Including those provided to participants or used in intervention delivery or in training of intervention providers. Provide information on where the materials can be accessed (e.g. online appendix, URL).*** |  |
|  | - Information explaining the No Gap Joint Replacement Pilot Program can be accessed online from the websites of participating providers.   For example: <https://www.nexushospitals.com.au/no-gap-surgery-for-patients-requiring-joint-replacement/>   - Information about the procedure, risks and benefits are provided during preoperative consultations with health professionals. - Patients also receive a written information booklet from their health insurer with details of the program prior to their surgery. |  |
| **WHAT procedures** | ***Describe each of the procedures, activities, and/or processes used in the intervention, including any enabling or support activities*** |  |
|  | **Learning about the short-stay care pathway and referral procedure**  Patients may learn of the program and access referral into the short-stay care pathway via multiple avenues, including:   - Primary care physician   - who then refers directly to surgeon (may, or may not, be aware of ‘no gap’ option) - Participating hospital   - by contacting participating hospital directly (via website or telephone) to request information about the short-stay care pathway and to request the contact details of the participating orthopaedic surgeons - Health insurer   - by responding to advertising and contacting the health insurer directly who then provides a list of participating surgeons and hospitals - Physiotherapy practice   - if known to the participating physiotherapy practice who provide with information about the pathway and participating hospitals / surgeons - Orthopaedic surgeon   - due to an existing relationship or word-of-mouth referral to surgeon   Due to the variety and variation of access to the model of care patients may present to initial pre-surgical consults with differing understanding of the model and perspective on model priorities. That is, some patients may prioritise the ‘no gap’ aspect whilst others seek a ‘short stay’ in hospital.  **Pre-operative consultation with orthopaedic surgeon**   - Conducts a comprehensive clinical assessment to determine patient eligibility for short-stay care pathway. - Assessments include use of ASA^^[[1]](#footnote-2)^^ and RAPT^^[[2]](#footnote-3)^^. Patients require ASA score of 1 (ASA 2 scores may be accepted, depending on the individual) to predict physical condition of an individual prior to anaesthetic. - If patient is eligible, surgeon provides education about the arthroplasty, short-stay care pathway and sets patient expectations about recovery plan   **Prehabilitation ‘joint school’**   - Prehabilitation educational seminar (also known as “Joint School”) provided in-person is a dedicated team-based event carried out with the purpose of encouraging patient-surgeon interaction. - The seminar is co-facilitated by physiotherapy and nursing staff. - The seminar helps to inform patients and their family/partner/support person about the arthroplasty surgery, perioperative care, anaesthetics, anticipated challenges, recovery, rehabilitation, and planned hospital length of stay. - This seminar provides an opportunity to set expectations and provide avenues of self-care and self-management to promote a comprehensive recovery from the surgical stress. - The session includes education about pain relief, ice machines, and gait aids.   **Preoperative assessment pre-admission clinic with Nurse Practitioner**   - Provides information regarding the anaesthetic plan and assists patient to complete a general health assessment and anaesthetic assessment which is passed on to the anaesthetist - Shows patient around the hospital wards and recovery areas - Orders pre-operative tests including blood test, electrocardiogram (ECG), and x-ray - Provides information regarding post-op medication - Ideally conducted 4 weeks prior to surgery although sometimes occurs 1-2 weeks prior to surgery due to delay in receiving referral   **Perioperative consultations / assessments:**   - Anaesthetist completes individual perioperative assessment. - Physiotherapy conduct 1:1 preoperative assessment, in-person onsite at the participating hospital to prescribe an individualised prehabilitation exercise program. - Ideally conducted 4 weeks prior to surgery although sometimes occurs 1-2 weeks prior to surgery due to delay in receiving referral   **Home Support Services (HSS) / Rehab in the Home (RITH)**   - Referral to home support services (HSS) received from orthopaedic surgeon/practice manager or health insurer concierge. - Case manager or a registered nurse conducts personal initial contact over the phone prior to patient admission to assess suitability for rehabilitation in the home (RITH). - Screening involves Occupational Health & Safety check, pre-morbid function, social support, and goal setting. - HSS and RITH appointments (nursing and physiotherapy) scheduled at this time. - Additional supports which may be required (e.g., domestic support, meal provision) are identified during this screening call. |  |
| **WHO PROVIDED** | ***For each category of intervention provider (e.g., psychologist, nursing assistant) describe their expertise, background and any specific training given*** | |
|  | **Outpatient consultation**   - Orthopaedic surgeon   **Preoperative assessment**   - Anaesthetist - Clinical Nurse Practitioner - Occupational therapist - Orthopaedic surgeon - Physiotherapist   **In hospital surgical intervention and recovery**   - Anaesthetist - Hospital Chief Executive Officer / Director of Nursing Services - Nursing staff - Nurse practitioner / pathway coordinator - Orthopaedic surgeon - Physiotherapist   **Post-hospital outpatient care at home**   - Registered nurse - Physiotherapist | |
| 1. **HOW** | ***Describe the modes of delivery (e.g. face-to-face or by some other mechanism, such as internet or telephone) of the intervention and whether it was provided individually or in a group****.* | |
|  | - Modes of delivery included individual face-to-face consultations at most stages of the intervention. - Some consultations were adapted due to COVID-19 restrictions and offered virtually via telehealth (phone or video conference call). - The only exception was the prehabilitation ‘joint school’ which was offered as in-person group sessions. If participants were unable to attend that the group session at the scheduled time, an individual session was offered. | |
| 1. **WHERE** | ***Describe the type(s) of location(s) where the intervention occurred, including any necessary infrastructure or relevant features.*** | |
|  | - Pre-operative consultations occurred at the surgeon’s private consulting rooms or onsite at the participating hospital. - In-patient hospital stay occurred in a small, metropolitan hospital. - Post-operative appointments typically occurred in the patient’s own home. Although there was some flexibility, for example, on occasions the physiotherapist met the patient at their workplace for a rehabilitation session.   **Additional features:**   - Patients hired or purchased crutches at their own expense (approximately $50 deposit which is refunded when equipment is returned) from an external hire company. - Patients hired other aids i.e., over toilet frame (OTF) also at their own expense from an external hire company. - Patients hired an ‘Ice Man’ machine to use at home during their outpatient recovery i.e., <https://www.betterbraces.com.au/iceman-clear3-cold-therapy-unit> | |
| 1. **WHEN and HOW MUCH** | ***Describe the number of times the intervention was delivered and over what period of time including the number of sessions, their schedule, and their duration, intensity or dose.*** | |
|  | **Preadmission**   - 1 x Initial consultation with orthopaedic surgeon - 1 x Joint school education session - 1 x Pre-admission appointment at hospital with nurse practitioner and physiotherapist   **During short acute inpatient admission**   - 1 x pre-surgical review by orthopaedic surgeon - 1 x pre-surgical review by anaesthetist - Arthroplasty surgery (typically 1-2 hours) - Intraoperative and post-operative nursing care - Physiotherapy assessment on ward usually within 4 hours post-operative and prior to discharge - 1 x post-operative review by orthopaedic surgeon - Discharge reviews and discharge home typically after 1 night admission   **Home-based rehabilitation**   - 1 x in-person nursing review within ~5 days post discharge home - Weekly telephone ‘welfare’ checks by nursing ~4 weeks - Twice weekly physiotherapy sessions for ~2 weeks - Weekly physiotherapy sessions for next ~ 4 weeks - Home-based rehabilitation ceases at ~ 6 weeks post arthroplasty - Final consultations with orthopaedic surgeon   - Typically, review at ~6-8 weeks post arthroplasty   - Re-review at ~12 months   - Ideally, ongoing annual reviews until 5 years | |
| 1. **TAILORING** | ***If the intervention was planned to be personalised, titrated or adapted, then describe what, why, when, and how.*** | |
|  | **Short-stay care pathway:**   - Patients receive a phone call from the hospital pathway coordinator one week post discharge to check on their recovery. Further follow up phone calls are made if needed (e.g., if the patient reports concerns during the initial phone call). The hospital pathway coordinator can further tailor the post-operative care if indicted during this phone call (e.g., initiate a referral for additional support services). - Anaesthetics (intraoperative and postoperative) protocol for enhance recovery largely follows latest evidence, however, this is tailored based on individual’s medical history, height, weight et cetera. - Physiotherapy care is tailored as indicated (e.g., gait aid prescription, exercise prescription). - Nursing care is tailored as indicated (e.g., if patient develops post-op infection additional services and reviews can be arranged. - Planned discharge date and/or destination can be altered if indicated during post-operative assessments by surgeon, physiotherapist, or nursing staff.     **Financial arrangements:**   - Patients pay an excess on their private health insurance which differs depending on their insurance plan. - Patients do not incur out-of-pocket fees for the hospital stay or surgery. - Patients may incur additional fees (i.e., for equipment hire) but this is disclosed to patients in advance at time of gaining financial consent. - Occasionally patients will require ‘extra’ services during their recovery period (e.g., additional physiotherapy sessions or investigative ultrasound). If the hospital administrators are aware of this they will pay on behalf of the patient. However, if patients seek care privately without notifying the hospital (i.e., via their general practitioner) they may pay out of the pocket for these ‘extra’ services. | |
| 1. **MODIFICATIONS** | ***If the intervention was modified during the course of the study, describe the changes (what, why, when, and how).*** | |
|  | N/A – retrospective study (after completion of short-stay care pathway) | |
| 1. **HOW WELL - PLANNED** | ***If intervention adherence or fidelity was assessed, describe how and by whom, and if any strategies were used to maintain or improve fidelity, describe them*** | |
|  | N/A – complex care pathway therefore intervention fidelity not assessed  Attendance at joint school and pre-operative appointment is strongly encouraged as an essential component of the short-stay care pathway. | |
| 1. **HOW WELL – ACTUAL** | ***Actual: If intervention adherence or fidelity was assessed, describe the extent to which the intervention was delivered as planned.*** | |
|  | N/A | |

1. ASA stands for *American Society of Anesthesiologists* [↑](#footnote-ref-2)
2. RAPT stands for Risk Assessment and Predictor Tool [↑](#footnote-ref-3)
